# Influence of Refraction on Widefield Retinal and Choroidal Vascular Metrics in Optical Coherence Tomography Angiography in a Caucasian Population

**DOI:** 10.1167/tvst.15.9.20

**Published:** 2026-09-25

**Authors:** Charlotte Egbring, Sarah Kleemann, Moritz Fabian Danzer, Nicole Eter, Jens Julian Storp

**Affiliations:** 1 Department of Ophthalmology, University of Muenster Medical Center, Muenster, Germany; 2 Department of Ophthalmology, University Hospital Ludwigs-Maximilians-University (LMU), Munich, Germany; 3 Institute of Biostatistics and Clinical Research, University of Muenster, Muenster, Germany

**Keywords:** widefield swept-source optical coherence tomography angiography (WF-SS-OCT-A), myopia, hyperopia, refraction, AngioTool

## Abstract

**Purpose:**

Refraction-related differences in retinal and choroidal vascular metrics have been reported using optical coherence tomography angiography (OCTA), primarily within the central fundus. This study aimed to establish normative vascular reference values for the retina and choroid using widefield swept-source OCTA (WF-SS-OCTA), stratified by spherical equivalent (SE), to characterize associations between refractive status and OCTA-derived vascular measurements extending beyond the central fundus.

**Methods:**

In this prospective single-center study, healthy volunteers underwent WF-SS-OCTA at the University Hospital Münster, Germany. Vascular metrics of the superficial capillary plexus (SCP) and the choroid were quantified using AngioTool software. Associations between SE and vascular metrics were analyzed using linear quantile mixed models accounting for inter-eye dependency and sex-specific effects.

**Results:**

A total of 414 eyes from 216 participants were analyzed. Exploratory analyses suggested an inverse relationship between SE and vascular endpoints in the SCP. In sex-stratified analyses, higher SE tended to be associated with increased flow density, vessel length, and junction counts in men, whereas junction counts tended to decrease in women. In the choroid, no clear associations were observed in the overall cohort; however, exploratory sex-specific analyses indicated potential associations between increasing SE and reduced junction density and total vessel length in women. Significant sex-related differences were observed for baseline junction counts and SE-related total vessel length trends.

**Conclusions:**

Refractive status may influence WF retinal and choroidal vascular metrics, with several parameters showing potential sex-specific patterns.

**Translational Relevance:**

The resulting refraction- and sex-stratified normative dataset may serve as a practical reference framework for the interpretation of WF-SS-OCTA measurements, whereas future studies incorporating axial length (AL) measurements are needed to refine anatomic interpretation.

## Introduction

Numerous studies based on optical coherence tomography (OCT) have consistently demonstrated refractive status-dependent variability in OCT-derived measurements, such as greater retinal and choroidal thickness in hyperopic compared with myopic eyes.^1^^–^^5^

In the 2010s, OCT angiography (OCTA) was introduced, allowing high-resolution visualization and quantification of the retinal microvasculature by detecting motion contrast from capillary blood flow.^6^ With regard to axial length (AL) and refractive status, a consistent body of evidence indicates reduced retinal flow density (FD) in myopic eyes compared with emmetropic or hyperopic eyes, affecting both the macular region^7^^–^^9^ and the peripapillary area.^7^^,^^8^^,^^10^^,^^11^ In contrast, findings on choroidal perfusion remain inconsistent, with some studies reporting reduced choroidal FD in myopia,^12^^,^^13^ whereas others observed no significant differences.^14^ Due to technical limitations, most existing studies have been confined to scan areas encompassing the macular and peripapillary regions, largely excluding the retinal periphery.

Recently, however, advances in imaging technology have resulted in the development of widefield swept-source OCTA (WF-SS-OCTA), with scan areas of up to 26 × 26 mm², substantially expanding the field of view to include the peripheral retina and choroid.^15^ Beyond disease-specific applications,^16^^–^^21^ a deeper understanding of physiological determinants of WF-SS-OCTA imaging is essential, as it remains unclear whether conclusions drawn from central OCTA observations can be extrapolated to other retinal and choroidal regions.

Although studies using WF-SS-OCTA have begun to address this issue,^22^^–^^25^ the recent availability of commercially available WF-SS-OCTA devices means that only a limited number of studies exist. Moreover, these studies have largely focused on myopia, neglecting hyperopia, and have assessed only a small set of vascular parameters, limiting their ability to provide a comprehensive understanding of global retinal and choroidal trends in relation to refractive status. In addition, recent studies indicate the presence of sex-dependent differences,^26^^,^^27^ which further restricts the explanatory power of individual studies.

To date, normative reference values for WF-SS-OCTA are lacking and currently accessible systems do not provide integrated databases. Consequently, quantitative vascular measurements obtained with this technology cannot yet be reliably interpreted in relation to physiological reference standards, limiting their usefulness in both research and clinical practice.

Therefore, the primary aim of the present study was to characterize WF-SS-OCTA vascular metrics in relation to refractive status and to establish refraction-stratified normative reference values for retinal and choroidal perfusion. By comparing eyes with varying refractive characteristics, this study seeks to establish a comprehensive quantitative reference dataset for retinal and choroidal perfusion in WF-SS-OCTA. As a secondary objective, potential interactions between refraction and sex were explored to assess whether sex-specific differences modulate refractive anatomy-related vascular patterns.

## Methods

### Design and Setting

This prospective observational study comprised healthy volunteers examined at the Department of Ophthalmology, University Hospital Münster, Germany, between December 2024 and November 2025. Prior to study participation, written informed consent was obtained from all individuals or their legal guardians. The study was approved by the Ethics Committee of the Medical Association of Westfalen-Lippe and the University of Münster (approval ID: 2024-332-f-S) and was conducted in accordance with the tenets of the Declaration of Helsinki.

### Patient Examination

All participants underwent a standardized ophthalmologic evaluation to confirm ocular health. This assessment included a detailed medical and ophthalmic history, slit-lamp examination of the anterior segment, funduscopic examination, objective and subjective refraction, and measurement of intraocular pressure. Only phakic eyes without a history of refractive surgery were included. Subjects were excluded if any ocular disease beyond refractive error was detected. Additional exclusion criteria were the presence of retinal or optic nerve pathology, diabetes mellitus, prior ocular surgery, or media opacities impairing image quality. To investigate associations across a clinically heterogeneous refractive range, no upper or lower refractive limits were applied.

After confirmation of eligibility, participants rested in a seated position for 5 minutes to reduce potential cardiovascular influences on ocular perfusion.^28^ Imaging was subsequently performed by an experienced examiner in a darkened, windowless examination room. Pharmacological pupil dilation was not applied, as mydriatic agents have been reported to influence vascular measurements obtained with WF-SS-OCTA.^29^

WF-SS-OCTA was conducted using the Canon Xephilio OCT-S1 system (Canon Inc., Tokyo, Japan), providing 23 × 20 mm scans with an 80-degree field of view (Fig. 1). All examinations were scheduled around midday (approximately 12 PM) to minimize diurnal variability.^30^ Retinal layer boundaries were delineated using the built-in automated segmentation software (Canon Ophthalmic Software Platform RX, version 5.0; RX Capture for OCT S1, RX Viewer, and RX Server), and the integrated Intelligent Denoise function was applied to improve vessel contrast and reduce noise. For each eye, a minimum of three scans was acquired. The scan with the highest quality index (QI) was selected for analysis; scans with motion artifacts or a QI < 6 were excluded. Both eyes of each participant were imaged and considered for inclusion in the analysis. Eyes that did not meet the predefined image quality criteria were excluded independently, whereas eligible fellow eyes remained in the study. In cases of identical QI values, one scan was selected at random. All analyses were restricted to the 23 × 20 mm acquisition protocol to ensure methodological consistency across all examinations.

**Figure 1.**
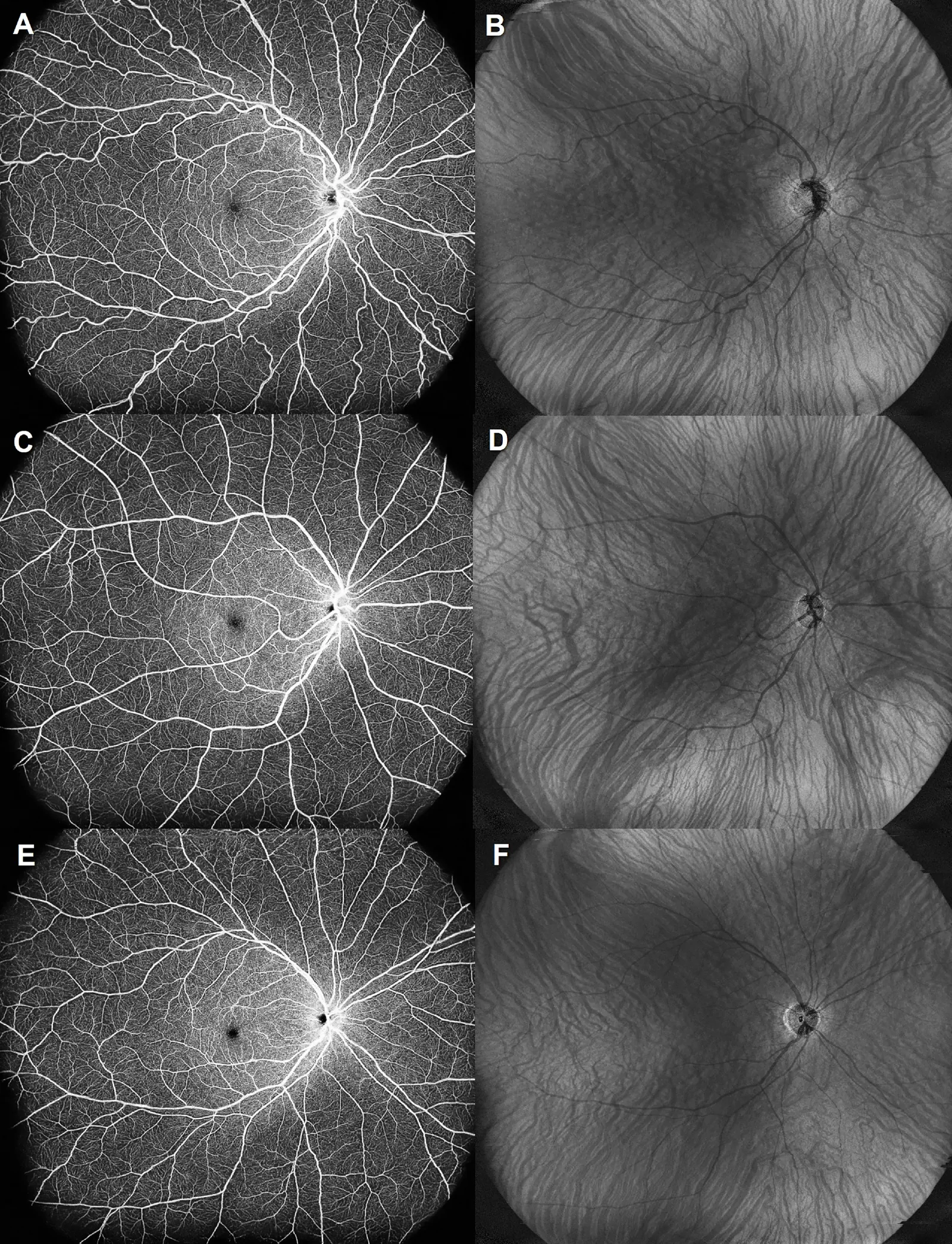
Examples for hyperopic, emmetropic, and myopic right eyes based on spherical equivalent (SE) in widefield swept-source optical coherence tomography angiography (WF-SS-OCTA) scans (23 × 20 mm^2^). *Left*: Superficial capillary plexus (SCP), *right*: choroid. (**A, B**) Hyperopic eye with a SE = +4 D, (**C, D**) emmetropic eye with a SE = −0.25 D, (**E, F**) myopic eye with an SE = −3.25 D.

As the Canon system does not offer built-in quantitative vascular analysis, image processing was performed using AngioTool software (National Cancer Institute [NIH], Gaithersburg, MD).^31^ Prior to analysis, choroidal images were inverted using Microsoft Paint (Microsoft, Redmond, WA) to ensure optimal vessel recognition. AngioTool software, originally introduced by Zudaire et al. for the calculation of vascular branching indices, was used to extract a comprehensive set of morphometric parameters describing the vascular network.

### Vascular Parameters

AngioTool software automatically quantified multiple descriptors of vascular architecture, including FD, number of vessel junctions, total vessel length, average vessel length, number of vessel endpoints, and lacunarity. FD reflects the proportion of vessel-related pixels relative to the total analyzed area and serves as an indicator of vascular density. Total vessel length corresponds to the cumulative length of all detected vessel segments, whereas average vessel length represents the mean distance between branching points. Endpoints were normalized to the scan area. Lacunarity, a parameter describing spatial heterogeneity, characterizes variations in pixel density between vascular and non-vascular regions.

To ensure reproducibility and comparability, segmentation parameters were defined prior to analysis and applied uniformly across all images. For retinal analysis, the lower threshold was set to 15, the upper threshold to 255, and vessel thickness to 3. For choroidal analysis, the lower threshold was set to 10, the upper threshold to 255, and vessel thickness to 10. These values were determined through an iterative optimization process involving multiple experienced investigators and were selected based on consensus following visual inspection to confirm accurate vessel delineation while minimizing background noise and segmentation artifacts. Once established, these parameters remained fixed for the entire dataset.

### Statistical Analysis

Baseline demographic and clinical characteristics are presented as absolute and relative frequencies for categorical variables and as medians with first and third quartiles for continuous variables.

Given that both eyes from individual participants were included, the clustered structure of the data was accounted for in all analyses. Although mixed-effects models are commonly applied in such settings, diagnostic evaluation revealed pronounced heteroskedasticity. Therefore, linear quantile mixed models^32^ were used, as they impose fewer distributional assumptions. In these models, spherical equivalent (SE) and sex were included as fixed effects, whereas participant identity was modeled as a random effect. Refractive status was represented by SE, calculated from the spherical and cylindrical refractive components. Astigmatism was not included as a separate covariate in the statistical models.

To describe population-level trends as well as SE- and sex-specific reference ranges, models were fitted for the 50th percentile (median) and the 5th and 95th percentiles. Unlike conventional regression approaches focusing on the conditional mean, these models estimate the relationship between covariates and specific quantiles of the outcome distribution. Results are presented as model coefficients and graphical visualizations for sex-stratified analyses. Because SE was used as the continuous predictor on the x-axis, the model intercept represents the estimated value of the respective vascular parameter at SE = 0 diopters (D), corresponding to emmetropia.

All statistical tests were 2-sided, with a significance level set at *P* < 0.05. Given the exploratory nature of the study, no correction for multiple testing was applied. Statistical analyses were performed using R software (version 4.5.2).^33^ Linear quantile mixed models were implemented using the *lqmm* package,^34^ and figures were generated with ggplot2.^35^

## Results

### Study Population Characteristics

A total of 414 eyes from 216 healthy participants were included in the final analysis. Eighteen eyes were excluded because the acquired scans did not meet the predefined image quality criteria (QI < 6 and/or relevant motion artifacts), whereas the corresponding fellow eyes fulfilled all inclusion criteria and were retained for analysis. Participant age ranged from 11 to 84 years. Baseline demographic and clinical characteristics are summarized in Table 1. The distribution of image QI and SE did not differ significantly between female and male participants.

**Table 1.** General Patient Characteristics


*N*, Eyes	414
*N*, patients	216
Median age, y	36 (27; 53)
Per group	
M	31 (26; 50)
F	38 (28; 53)
Gender, M: F	80 (37%): 136 (63%)
Study eye, R: L	207 (50%): 207 (50%)
Median visual acuity, logMAR	0.00 (0.00; 0.00)
Median spherical equivalent	0.00 (−0.88; 0.63)
Per group	
M	0.00 (−0.75; 0.63)
F	0.00 (−1.00; 0.63)
Median QI	7.00 (6.00; 7.00)

F, female; L, left; logMAR, logarithm of the minimum angle of resolution; M, male; *N*, number; QI, quality index; R, right.

Values are reported as absolute numbers and as median (25% and 75% quartile).

The SE ranged from −7 D to 5 D. According to clinically relevant refractive categories, 124 eyes (30%) were hyperopic (> +0.50 D), 193 eyes (47%) were emmetropic (−0.50 to +0.50 D), 68 eyes (16%) had low myopia (< −0.50 to −3.00 D), 26 eyes (6%) had moderate myopia (≤ −3.00 to −6.00 D), and 3 eyes (1%) had high myopia (< −6.00 D).

### Regression Analysis

Across the entire study population, regression analyses identified a significant inverse association between SE and the number of endpoints in the superficial capillary plexus (SCP). No significant relationships were observed between SE and any other SCP vascular metrics when the cohort was analyzed as a whole.

Sex-specific analyses revealed distinct patterns. In male participants, increasing SE was associated with a significant rise in FD, as well as in total and average vessel length within the SCP. Each 1-diopter increase in SE was associated with a 0.26 percentage-point increase in SCP FD, corresponding to an estimated difference of approximately 1.3 percentage points across a 5-D refractive range. In contrast, the number of endpoints decreased significantly with higher SE exclusively in men. The number of vessel junctions showed a significant association with SE in both sexes; however, the direction of change differed, with junction counts increasing in men and decreasing in women. Lacunarity did not demonstrate a significant relationship with SE in either sex.

Direct comparisons between male and female participants demonstrated statistically significant sex-dependent differences in the number of junctions, total, and average vessel length. For all three parameters, values increased with higher SE in men, whereas a decreasing trend was observed in women. Detailed numerical results are provided in Table 2, and corresponding graphical representations are shown in Figure 2.

**Table 2.** Values and 95% Confidence Intervals for Quantile Regression Analysis of the Median

Parameter	Sex	Intercept Value (LB; UB)	SE (LB; UB)	*P* Value, SE
FD (%)	B	43.38 (43.17; 43.59)	0.10 (0.00; 0.21)	0.06
	M	43.26 (42.85; 43.67)	0.26 (0.05; 0.47)	**0.02**
	F	43.29 (43.01; 43.57)	0.05 (−0.11; 0.22)	0.53
	*P*, sex	0.91	0.13	
NOJ, *n*	B	11,281 (11,157; 14,406)	1 (−51; 54)	0.96
	M	11,285 (11,120; 11,449)	123 (28; 218)	**0.01**
	F	11,174 (11,022; 11,325)	−70 (−136; −3)	**0.04**
	*P*, sex	0.27	**<0.01**	
TVL, µm	B	240,444 (239,055; 241,833)	−90 (−912; 732)	0.83
	M	241,427 (239,163; 243,692)	1,687 (46; 3,329)	**0.04**
	F	240,383 (238,744; 242,021)	−547 (−1,302; 207)	0.15
	*P*, sex	0.45	**0.01**	
AVL, µm	B	370 (357; 383)	5 (−3; 12)	0.23
	M	375 (357; 393)	11 (3; 21)	**<0.01**
	F	372 (359; 386)	−1 (−8; 6)	0.79
	*P*, sex	0.83	**0.02**	
NOE, *n*	B	4,667 (4,624; 4,710)	−35 (−59; −11)	**<0.01**
	M	4,704 (4,645; 4,763)	−40 (−80; −1)	**0.05**
	F	4,636 (4,578; 4,695)	−29 (−64; 6)	0.11
	*P*, sex	0.07	0.29	
Lac	B	0.04 (0.04; 0.04)	0.00 (0.00; 0.00)	0.21
	M	0.04 (0.04; 0.05)	0.00 (0.00; 0.00)	0.11
	F	0.04 (0.04; 0.04)	0.00 (0.00; 0.00)	0.94
	*P*, sex	0.56	0.14	

AVL, average vessel length; B, both sexes; F, female; FD, flow density; Lac, lacunarity; LB, lower boundary; M, male; *n*, number; NOE, number of endpoints; NOJ, number of junctions; SE, spherical equivalent; TVL, total vessel length; UB, upper boundary.

*P* values < 0.05 are highlighted in bold. *P* values are displayed for sex- and SE-comparisons, respectively.

**Figure 2.**
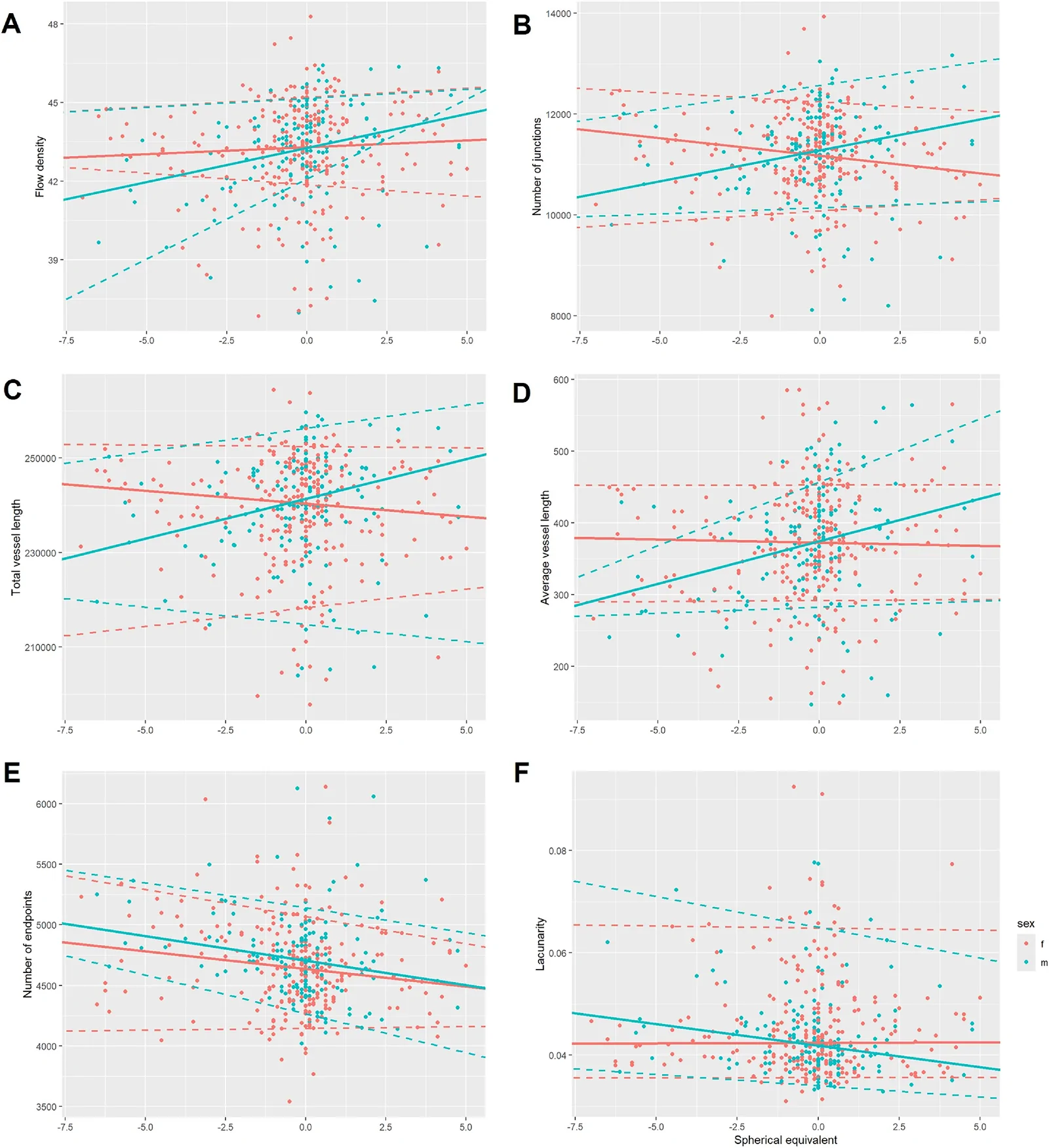
Scatter plots of AngioTool parameters of the superficial capillary plexus (SCP) in relation to spherical equivalent (SE). (**A**) Scatter plot of flow density (FD; %) and SE. (**B**) Scatter plot of number of junctions (NOJs) and SE. (**C**) Scatter plot of total vessel length (TVL; µm) and SE. (**D**) Scatter plot of average vessel length (AVL; µm) and SE. (**E**) Scatter plot of number of endpoints (NOEs) and SE. (**F**) Scatter plot of lacunarity (Lac) and SE (m = male [*blue*]; f = female [*red*]). Note that the regression line of the median is continuous, whereas the regression lines of the 0.05 and 0.95 quantiles are dashed.

Regarding the choroid, analyses of the entire cohort revealed no significant associations between SE and any vascular metrics. In sex-stratified analyses, however, increasing SE was significantly associated with a reduction in both the number of junctions and total vessel length in female participants. Comparisons between sexes demonstrated a significantly higher intercept for the number of junctions in men, as well as a significant sex-specific difference in the relationship between SE and total vessel length, which increased in men but decreased in women. Detailed results are summarized in Table 3, with graphical illustrations presented in Figure 3.

**Table 3.** Values and 95% Confidence Intervals for Quantile Regression Analysis of the Median

Parameter	Sex	Intercept Value (LB; UB)	SE (LB; UB)	*P* Value, SE
FD (%)	B	44.48 (44.24; 44.72)	0.08 (−0.08; 0.23)	0.33
	M	44.58 (44.25; 44.86)	0.06 (−0.12; 0.23)	0.54
	F	44.35 (44.01; 44.68)	0.00 (−0.20; 0.20)	0.99
	*P*, sex	0.34	0.67	
NOJ, *n*	B	886 (871; 900)	−4 (−10; 3)	0.24
	M	911 (893; 929)	−5 (−15; 6)	0.41
	F	861 (848; 874)	−7 (−13; −1)	**0.02**
	*P*, sex	**<0.01**	0.67	
TVL, µm	B	82,854 (82,304; 83,404)	−176 (−376; 25)	0.09
	M	83,681 (83,310; 84,053)	26 (−148; 201)	0.77
	F	82,582 (82,147; 83,018)	−258 (−474; −41)	**0.02**
	*P*, sex	0.45	**0.05**	
AVL, µm	B	514 (502; 528)	−4 (−13; 5)	0.33
	M	532 (506; 557)	−4 (−16; 8)	0.50
	F	512 (496; 529)	0 (−10; 10)	0.96
	*P*, sex	0.20	0.61	
NOE, *n*	B	864 (842; 887)	2 (−8; 12)	0.75
	M	871 (847; 894)	3 (−10; 17)	0.62
	F	858 (835; 882)	−2 (−14; 10)	0.76
	*P*, sex	0.36	0.56	
Lac	B	0.09 (0.09; 0.09)	0.00 (0.00; 0.00)	0.78
	M	0.09 (0.08; 0.09)	0.00 (0.00; 0.00)	0.68
	F	0.09 (0.09; 0.09)	0.00 (0.00; 0.00)	0.94
	*P*, sex	0.63	0.75	

*P* values < 0.05 are highlighted in bold. *P* values are displayed for sex- and SE-comparisons, respectively.

**Figure 3.**
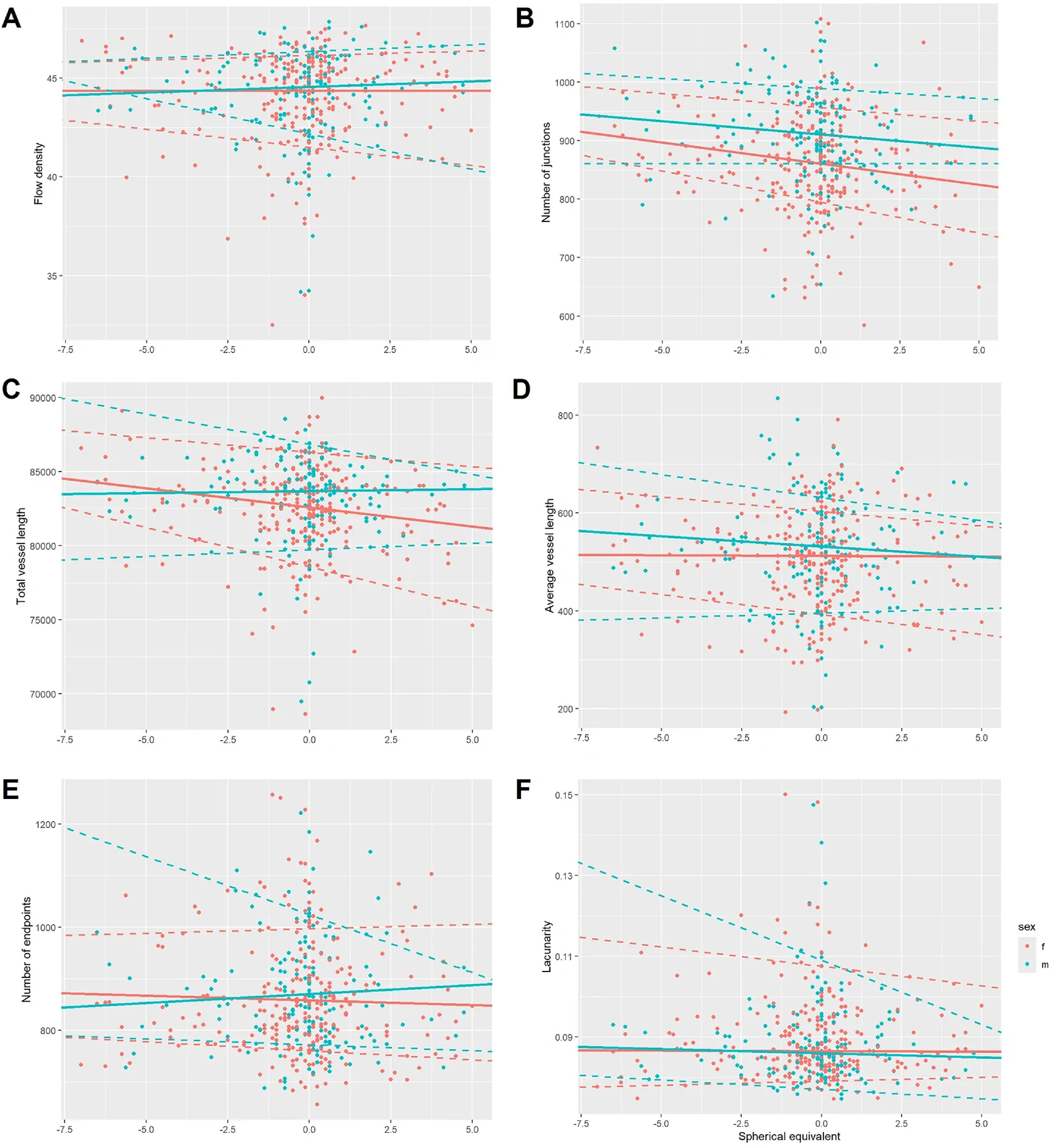
Scatter plots of AngioTool parameters of the choroidal vasculature in relation to spherical equivalent (SE). (**A**) Scatter plot of flow density (FD; %) and SE. (**B**) Scatter plot of number of junctions (NOJs) and SE. (**C**) Scatter plot of total vessel length (TVL; µm) and SE. (**D**) Scatter plot of average vessel length (AVL; µm) and SE. (**E**) Scatter plot of number of endpoints (NOEs) and SE. (**F**) Scatter plot of lacunarity (Lac) and SE (m = male [*blue*]; f = female [*red*]). Note that the regression line of the median is continuous, whereas the regression lines of the 0.05 and 0.95 quantiles are dashed.

## Discussion

The present study provides a comprehensive quantitative overview of retinal and choroidal vascular metrics obtained by WF-SS-OCTA in a large cohort of healthy subjects across a broad refractive range. By including eyes with myopic, emmetropic, and hyperopic refraction, this work aimed to characterize physiological variability across a range of refractive states and establish normative reference values. Because AL measurements were not available, analyses were based on refractive status as represented by SE, enabling the investigation of refraction-associated physiological variability in retinal and choroidal perfusion.

Across the entire study population, no significant association was observed between SE and FD in the SCP. This finding contrasts with numerous earlier OCTA studies reporting reduced retinal FD in myopic eyes compared with emmetropic or hyperopic eyes.^7^^–^^11^ However, those studies relied on conventional macula-centered OCTA scans with limited fields of view, limiting comparability.

More recent WF-SS-OCTA investigations covering larger fields of view have reported lower SCP FD in both the central and peripheral retina of myopic eyes.^22^^–^^25^ Gu et al. compared the SCP in 57 low myopic and 34 high myopic Asian individuals (SE <−6 D). Using the built-in algorithm of the Bmizar BM-400K system (Toward Pi Medical Technology, Ltd., Beijing, China), they demonstrated a significant reduction in FD in the latter group, which correlated significantly with refractive error.^22^ These findings were confirmed and extended by Wang et al., who examined 506 Asian eyes, including emmetropic controls, and further stratified high myopic eyes according to AL below or above 28 mm. Using the built-in analysis software of the VG200 system (Svision Imaging, Ltd., Luoyang, China), they identified a negative correlation between AL and peripheral SCP FD.^23^

When comparing these results with those of the present study, differences in ethnic composition, study populations, imaging platforms, and analytical approaches must be taken into account. Notably, the present cohort consisted exclusively of Caucasian participants, and both the imaging device and post-processing algorithms differed from those used in the aforementioned studies, which may have contributed to divergent findings.

Beyond potentially contributing to differences between studies, the imaging platform and analytical workflow should also be considered when interpreting the normative values reported here. The absolute values presented in this study should be regarded as specific to the imaging and analytical approach used and may not be directly transferable to other OCTA platforms or software solutions.

In addition, it is important to note that the present study demonstrated a decreasing trend in SCP FD with lower SE in both sexes, which reached statistical significance in men (*P* = 0.02). In the studies by Gu et al. and Wang et al., the sex distribution differed substantially between comparison groups. For example, Wang et al. reported a female proportion of 52% in the highly myopic group compared with 65% in the emmetropic control group.^23^ In light of the present findings, it is possible that differences in sex distribution between study groups may have influenced the reported associations. However, the extent to which sex composition contributed to these findings cannot be determined from the available data and likely represents only one of several methodological differences between studies.

Across the entire cohort, the number of endpoints in the SCP demonstrated a significant inverse association with SE, indicating a higher number of terminal vessel segments in more myopic eyes. This finding may reflect increased microvascular fragmentation or altered vascular remodeling potentially associated with structural characteristics frequently observed in myopic eyes, which is consistent with previously reported structural retinal changes, including retinal thinning in myopic eyes.^2^^–^^4^ Sex-specific analyses revealed that this association was again driven by male participants, in whom the decrease in the number of endpoints with increasing SE reached statistical significance.

Additional SCP parameters further emphasized sex-dependent refractive patterns. Although no consistent trend was observed in the pooled analysis, junction counts as well as total and average vessel length increased significantly with higher SE in men but decreased in women. Direct comparisons confirmed significant differences in regression slopes between sexes for all three metrics. These opposing trends indicate that the association between refractive status and retinal microvascular metrics may differ between men and women. One possible explanation may relate to the greater retinal thickness commonly observed in men,^36^^–^^38^ which could be associated with higher baseline vascular complexity and, consequently, with more pronounced refraction-related remodeling. However, the present study was not designed to investigate underlying mechanisms and any biological interpretation remains speculative.

Previous studies investigating sex-related differences in retinal OCTA parameters have yielded inconsistent results.^39^^–^^47^ Several studies have reported higher retinal FD in men^39^^,^^40^ and larger foveal avascular zone (FAZ) areas in women^40^^–^^44^ in central retinal imaging. However, other authors have found no significant sex-specific differences in retinal vascular parameters.^45^^–^^47^ The present findings suggest that refractive anatomy and its interaction with sex may contribute to these discrepancies and highlight the importance of stratified analyses when establishing normative reference values.

Compared with the SCP, choroidal vascular parameters demonstrated fewer and weaker associations with SE, particularly in the overall cohort. This finding aligns with the heterogeneous and often conflicting literature on refractive influences on choroidal perfusion.^12^^–^^14^^,^^39^^,^^48^^,^^49^

No significant association between SE and choroidal FD was observed in either the pooled or sex-stratified analyses. This contrasts with recent WF-SS-OCTA studies reporting increased choroidal FD in myopic eyes.^24^^,^^25^ Si et al. examined 33 emmetropic and low myopic eyes, 39 moderately myopic eyes, and 54 highly myopic eyes, reporting not only a progressive decline in SCP FD with increasing myopia severity but also a significant increase in choroidal perfusion.^24^ Similarly, Park et al. compared 32 myopic eyes with 14 emmetropic control eyes and found that increasing myopia was negatively correlated with retinal FD but positively correlated with choroidal perfusion, suggesting partial compensatory perfusion at the choroidal level.^25^ However, both studies differed substantially from the present work in terms of cohort composition, analytical parameters, and outcome measures. Si et al. assessed choroidal FD across multiple subregions,^24^ whereas Park et al. relied on the choroidal vascularity index (CVI) rather than FD itself,^25^ limiting direct comparability.

In the present study, sex-stratified analyses revealed a significant increase in choroidal junction counts and total vessel length with decreasing SE in women. It may be speculated that changes in CVI are more closely reflected by these parameters than by choroidal FD alone. This again underscores the need for standardized analytical protocols and target parameters to ensure comparability across studies and to facilitate meaningful interpretation of results.

Regarding the number of choroidal vessel junctions, men exhibited a significantly higher intercept, indicating a generally more interconnected choroidal vascular network. These findings are consistent with previous reports describing both greater choroidal thickness^37^^,^^50^^–^^52^ and higher peripheral choroidal perfusion in men.^26^^,^^27^

Lacunarity did not show a significant association with SE in either the SCP or the choroid, nor in the overall or sex-stratified analyses. This suggests that the spatial heterogeneity and gap distribution of the retinal and choroidal capillary networks are relatively stable across refractive states and between sexes. As lacunarity reflects the textural organization rather than the density or extent of vessels, it may represent a robust parameter that is less susceptible to physiological variability and therefore potentially useful for detecting pathological alterations.

Overall, the present study highlights substantial physiological variability in WF-SS-OCTA-derived vascular metrics related to refractive status. These findings have important clinical implications, as pooled normative values may obscure meaningful physiological differences and potentially lead to misinterpretation of quantitative vascular measurements. The data presented here represent an essential step toward establishing robust, refraction-aware normative reference datasets for WF-SS-OCTA, which are currently lacking in commercially available systems.

### Limitations

Important limitations of the present study should be noted.

First, previous research has demonstrated that OCTA-derived vascular parameters are influenced by ethnicity.^53^^,^^54^ As the present study exclusively included participants of Caucasian origin, the generalizability of the reported normative values and refractive-specific associations to other ethnic populations may be limited. Validation in more ethnically diverse cohorts is therefore warranted. Moreover, although no upper age limit was applied during recruitment and participants covered a broad age range, the median age was relatively young. As a result, the present cohort may not fully reflect the demographic characteristics and vascular risk profile of populations commonly investigated in oculomics research, which often include a higher proportion of older individuals and subjects with age-related systemic vascular risk factors. Because retinal and choroidal vascular parameters are known to be influenced by aging and systemic vascular health, the generalizability of the present findings to older populations should be interpreted with caution. Future studies including larger numbers of participants above 50 years of age will be important to determine whether the observed refractive status-dependent associations remain consistent across age groups and in cohorts more representative of typical oculomics populations. Furthermore, although age and sex were evaluated separately, potential age-by-sex interaction effects were not specifically investigated.

Second, the range of refractive errors included in the present cohort was relatively limited, particularly with respect to extreme myopia. Although no refractive limits were imposed during recruitment, the refractive distribution was centered around emmetropia, with 47% of eyes classified as emmetropic. Whereas this allowed for a robust characterization of vascular parameters across common refractive states, it may have precluded the detection of vascular alterations that occur predominantly in eyes with very high refractive errors. Previous studies have included eyes with ALs exceeding 28 mm, corresponding to refractive errors well beyond −10 D.^23^ The absence of such highly myopic eyes in the present study may therefore explain why certain alterations observed in these cohorts were not detected. Future studies should include a broader spectrum of refractive errors and axial elongation to more comprehensively assess vascular changes at the extremes of myopia.

Third, although WF-SS-OCTA enables visualization of a substantially larger retinal area than conventional OCTA, the accuracy of vessel segmentation and quantification in the far periphery has not yet been validated to the same extent as central macular measurements. Potential image distortions, reduced signal quality, and segmentation errors in peripheral regions may have influenced the calculated vascular metrics. Therefore, peripheral findings should be interpreted cautiously until standardized validation studies for wide-field vascular quantification become available.

Finally, AL was not measured in the present study. Although SE is frequently used as a clinical indicator of refractive status and is often correlated with AL at the population level, it does not directly reflect ocular size and cannot fully account for anatomic variation in globe geometry. Consequently, the present study cannot distinguish refractive effects from AL-related effects. Furthermore, ocular magnification correction could not be performed because established correction approaches require direct AL measurements. Therefore, part of the observed differences in vessel length-related and topology-based vascular metrics may reflect scaling effects rather than true biological vascular differences. Future studies incorporating ocular biometry and magnification-corrected analyses are required to validate and extend the present findings.

## Conclusions

This study establishes comprehensive normative data for retinal and choroidal vascular metrics derived from WF-SS-OCTA in a large healthy cohort with diverse refractive characteristics.

Exploratory analyses identified associations between refractive status and WF-SS-OCTA-derived vascular metrics, particularly within the SCP, whereas choroidal parameters showed potential sex-related patterns. These findings emphasize the importance of including refractive status in the interpretation of WF-SS-OCTA measurements and provide an important reference framework for future clinical and research applications of WF-SS-OCTA.
